# Oroxin A alleviates early brain injury after subarachnoid hemorrhage by regulating ferroptosis and neuroinflammation

**DOI:** 10.1186/s12974-024-03099-3

**Published:** 2024-05-03

**Authors:** Junhui Chen, Zhonghua Shi, Chunlei Zhang, Kun Xiong, Wei Zhao, Yuhai Wang

**Affiliations:** 1 Department of Neurosurgery, 904 th Hospital of Joint Logistic Support Force of PLA, Wuxi Clinical College of Anhui Medical University, No. 101 Xingyuan North Road, Liangxi District, Wuxi, 214044 Jiangsu Province China; 2 Department of Human Anatomy and Neurobiology, School of Basic Medical Science, Central South University, Changsha, Hunan Province China

**Keywords:** Oroxin A, Nrf2, Subarachnoid hemorrhage, Ferroptosis, Neuroinflammation

## Abstract

**Background:**

Subarachnoid hemorrhage (SAH), a severe subtype of stroke, is characterized by notably high mortality and morbidity, largely due to the lack of effective therapeutic options. Although the neuroprotective potential of PPARg and Nrf2 has been recognized, investigative efforts into oroxin A (OA), remain limited in preclinical studies.

**Methods:**

SAH was modeled in vivo through filament perforation in male C57BL/6 mice and in vitro by exposing HT22 cells to hemin to induce neuronal damage. Following the administration of OA, a series of methods were employed to assess neurological behaviors, brain water content, neuronal damage, cell ferroptosis, and the extent of neuroinflammation.

**Results:**

The findings indicated that OA treatment markedly improved survival rates, enhanced neurological functions, mitigated neuronal death and brain edema, and attenuated the inflammatory response. These effects of OA were linked to the suppression of microglial activation. Moreover, OA administration was found to diminish ferroptosis in neuronal cells, a critical factor in early brain injury (EBI) following SAH. Further mechanistic investigations uncovered that OA facilitated the translocation of nuclear factor erythroid 2-related factor 2 (Nrf-2) from the cytoplasm to the nucleus, thereby activating the Nrf2/GPX4 pathway. Importantly, OA also upregulated the expression of FSP1, suggesting a significant and parallel protective effect against ferroptosis in EBI following SAH in synergy with GPX4.

**Conclusion:**

In summary, this research indicated that the PPARg activator OA augmented the neurological results in rodent models and diminished neuronal death. This neuroprotection was achieved primarily by suppressing neuronal ferroptosis. The underlying mechanism was associated with the alleviation of cellular death through the Nrf2/GPX4 and FSP1/CoQ10 pathways.

**Supplementary Information:**

The online version contains supplementary material available at 10.1186/s12974-024-03099-3.

## Introduction

Cerebrovascular diseases often lead to the occurrence of subarachnoid hemorrhage (SAH), which is associated with high rates of fatality and morbidity. This condition also results in unfavorable outcomes, especially in individuals with hypertension. Incidence rates in Western nations range from 6.2 to 10 per 100,000 individuals [1–3]. The primary factors leading to unfavorable outcomes in patients with SAH are early brain injury (EBI) and cerebral vasospasm (CVS). Nevertheless, recent medical research has confirmed that although pharmacological interventions notably ameliorate CVS, they fail to show a beneficial effect on the overall prognosis following SAH [4]. Additionally, our prior clinical investigation also showcased this phenomenon [5]. Current studies point to a substantial impact of EBI following SAH, which appears to have a significant impact after SAH [6–8]. The underlying causes of EBI are varied and include biological processes, such as direct neuronal death, neuroinflammation, autophagic and apoptotic potential, and necroptosis [9–12]. Nevertheless, Zille et al. [13]. delineated that the impacts of inhibitors on caspase-dependent apoptotic capacity, protein or mRNA synthesis, mitophagy, autophagic potential, or parthanatos were insignificant in the context of intracerebral hemorrhage (ICH) both in vitro and in vivo. Conversely, agents that impede ferroptosis offer protection against the deleterious impacts of hemoglobin and hemin. However, the precise contribution of various forms of cell death to the toxicity resulting from SAH remains uncharacterized.

Extensive research has focused on ferroptosis, a recently identified process of cellular death. Dixon first reported this unique form of cell apoptosis in 2012, which is characterized by its dependence on iron-dependent programmed cell death. This particular cellular death pathway is especially crucial in neurons of the central nervous system (CNS) and holds promise in substantially diminishing the occurrence of neurodegenerative disorders, assuming successful prevention of iron-dependent cell necrosis [14]. Preventing intracellular calcium overload is essential, and inhibitors related to apoptosis prove to be ineffective in this context. The morphological characteristics of cell death typically involve the disappearance of mitochondrial cristae, prominent constriction of mitochondria, an increase in the thickness of the lipid bilayer, reduction in cell size, weakening of cell connections, and a gradual process of detachment. The primary features of biology encompass the malfunctioning of iron ions metabolism, reduction of glutathione (GSH) levels, accumulation of iron-dependent lipid reactive oxygen species (ROS), and the inhibition or reduced functioning of glutathione peroxidase 4 (GPX4) [15]. According to recent research, it has been verified that ferroptosis is prevalent in various CNS disorders and plays a role in the healing process, aging, tumors, ICH, and ischemia of the CNS [13, 14, 16].

In our prior investigation, it was discovered that netrin-1 can mitigate neuronal death by suppressing ferroptosis through the PPARγ/Nrf2/GPX4 pathway, the expression of Nrf2 and GPX4 mRNA is reduced, and neuronal ferroptosis is exacerbated by the PPARγ antagonist GW9662 [17]. According to Shin [18], the resistance of HNC cells to GPX4 inhibition was attributed to the activation of the Nrf2–ARE pathway, and reversing the resistance to ferroptosis in HNC was achieved by inhibiting this pathway. Nrf2 activation additionally facilitates iron retention, decreases cellular iron absorption, and restricts the generation of ROS. In many organisms and diseases, Dixon [19] examined the significance of ROS and iron as crucial triggers and facilitators of cell death (ferroptosis). The iron-dependent buildup of ROS is caused by the deactivation of the cystine-glutamate antiporter SLC7A11 and the depletion of glutathione. Furthermore, preliminary research has indicated that prompt treatment involving PPARγ agonists or PPARγ activation can significantly enhance neuronal repair and address functional impairments in cases of acute CNS disorders [17, 20–22]. Additionally, previous studies demonstrated that PPARγ activation can inhibit pro-inflammatory microglial responses, regulate microglial/macrophage M1/M2 polarization and alleviate neuroinflammation, then alleviate cognitive functions and EBI in chronic hypoperfusion-induced dementia, and traumatic brain injury [23–25]. M1/M2 polarization of microglia plays an important role in the neuroinflammatory response of the central nervous system, microglia M2 polarization can alleviate neuroinflammation and enhance hematoma clearance after experimental germinal matrix hemorrhage [26]. The use of PPARγ agonist medications, like rosiglitazone and pioglitazone, in clinical settings is controversial due to their potential to cause weight gain and an elevated risk of heart failure [27]. Hence, more effective, safer drugs with fewer side effects are urgently needed.

Oroxin A (OA), alternatively referred to as baicalein-7-O-glucoside, is a potent flavonoid compound derived from the traditional Chinese herb *Oroxylum indicum* (L.) Kurz (known as Mu Hu Die in Chinese). Historically, this herb has seen widespread use in the treatment of a variety of conditions, such as pharyngitis, acute and chronic bronchitis, coughs, and pertussis for centuries [28, 29]. OA has various bioactivities, such as triggering endoplasmic reticulum (ER) stress [28], suppressing pyroptosis, and functioning as an antioxidant [30]. Surprisingly, the pharmacological effects of OA are strongly linked to the stimulation of PPARγ. Nevertheless, there is a scarcity of reports on OA in preclinical investigations of SAH. The objective of this study was to elucidate the impact of OA on neuronal cell death and neurological function, as well as investigate the underlying molecular mechanisms following SAH. These findings could potentially enhance the advantages of targeting ferroptosis in SAH, lay the foundation for future drug development, and introduce novel clinical treatment alternatives.

## Experimental model and subject details

### Animals

The animal-based experimental protocols were approved by the Institutional Animal Care and Use Committee of Wuxi Clinical College of Anhui Medical University (Approval No. YXLL-2019-020). The procedures were in compliance with the *Guide for the Care and Use of Laboratory Animals* published by the National Institutes of Health (NIH) and adhered to the guidelines of Animal Research: Reporting of In Vivo Experiments, amendment of 1996, article 80 − 23. Male C57BL/6 mice (weighing 22–25 g; aged 8–10 weeks) were acquired from the Laboratory Animal Facility of Nantong University (Nantong, China). They were maintained in a controlled, pathogen-free environment at an ambient temperature of around 22 °C and under a 12-h light-dark cycle. The mice had unrestricted access to food and water and were utilized for in vivo experiments. Before the experiments, the animals were in a state of optimal health without prior medical interventions or exposure to any drug treatments.

### SAH model in mice

We previously described a protocol [31] for generating the mouse SAH model using the endovascular perforation technique. Briefly, anesthesia was administered to the animals through an intraperitoneal route utilizing pentobarbital sodium (50 mg/kg). To ensure optimal surgical conditions, a heating pad was employed to maintain the operative temperature at 37 ± 0.5 °C. A median incision in the neck was performed to access the left external carotid artery (ECA) and internal carotid artery (ICA). Following this, the left ECA was ligated and severed, creating a residual stump measuring 3 mm. A sharpened 5–0 monofilament nylon suture was introduced into the left ICA *via* the stump of the ECA to perforate the artery at the bifurcation of the anterior and middle cerebral arteries. To penetrate the bifurcation of the anterior and middle cerebral artery, the particular approach involved encountering resistance and subsequently pushing it an additional 2 mm. After removing the suture, the ICA was repercussed to induce SAH. Furthermore, sham group mice experienced identical processes without any perforation of the artery.

### Mortality and neurological deficits

Mortality was documented at 48 h after SAH. Additionally, the assessment of the severity of brain injuries was carried out using the neurological scoring system provided at the identical time interval [32]. In short, the assessment relied on an 18-point framework comprising six examinations that can be graded 0–3 or 1–3. These examinations encompassed spontaneous actions (0–3), symmetry of limb movements (0–3), body awareness (1–3), extension of forelimbs (0–3), climbing ability (1–3), and sensitivity to vibrissae touch (1–3). An impartial observer assessed all examinations without knowledge of the treatment conditions. The neurological score spans from 3 to 18, with a higher score denoting enhanced neurological functions [31].

### Brain water content

The standard wet-dry method, as previously explained [6, 17, 31], was used to assess the water content of the brain. collected for analysis at the 48-hour mark following SAH, where mice were euthanized and their entire brains were obtained. The brains were swiftly extracted and immediately separated into the left and right cerebral hemispheres, brain stem, and cerebellum, followed by an instantaneous measurement to ascertain their wet weight. After that, the specimens were subjected to dehydration at a temperature of 105 °C for a duration of 24 h to obtain the weight when completely dry. The water content of the brain was determined by first deducting the dry weight from the wet weight, then dividing this difference by the wet weight, and finally multiplying the quotient by 100% to obtain the percentage.

### Cultured cell line

HT22 cells from the hippocampus were acquired from the Bena Culture Collection located in China. In the incubation process, the cells were maintained at 37 °C in an environment comprising 5% CO_2_ using Dulbecco’s Modified Eagle’s Medium (DMEM). This medium was enriched with 10% FBS provided by Gibco (New Zealand) and 1% penicillin/streptomycin at 4 mg/ml from Sigma Aldrich. The tests were carried out with cell confluency of 60–80%. The authentication of HT22 cells was based on their visual morphology and susceptibility to toxicity triggered by erastin/hemin. Additionally, the cells utilized in the studies were restricted to a maximum of 25 passages. The plates utilized for the cultivation of cell lines were not coated.

### SAH model in HT22 cells

In the in vitro SAH experiments, cell death was initiated in hippocampal HT22 cells by subjecting them to hemin according to our previous study [17]. The dose-response ranged from 20 to 140 mmol/L, and this treatment lasted for 48 h. Hemin was dissolved using 0.1 M NaOH (Cat#H9039) provided by Sigma Aldrich. In this research, cells underwent treatment in the presence of designated compounds or sodium selenite (Sigma Aldrich) dissolved in ddH2O, using a concentration of 100 mmol/L hemin (LD50). Hemin was made ready and dissolved in a culture medium to a concentration of 100 mmol/L, then sterilized by filtering it through a 0.22-µm sterile filter. The assessment of cell viability was carried out 48 h following manipulation. Afterward, a warm 37 °C PBS wash was administered to the cells, and the evaluation of cell viability was conducted following the manufacturer’s recommendations, as specified by Promega. To replicate the conditions of SAH or ICH, neurons were stimulated with 100 mmol/L hemin for a duration of 48 h, as established in a previous investigation [17, 33].

### Drug administration

**Fig. 1:**
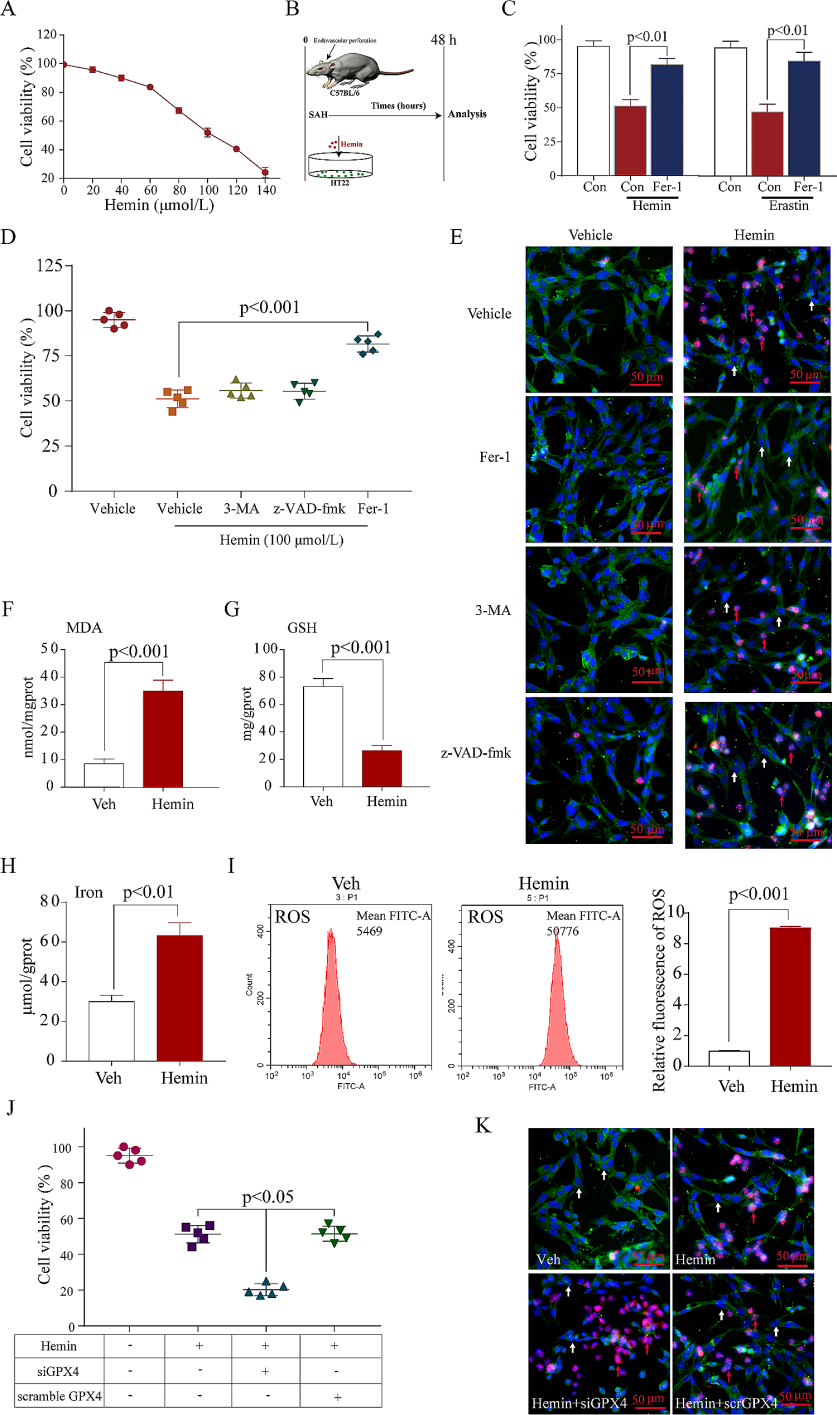
Ferroptosis plays a vital role in SAH and hemin-induced neuronal injury *in vitro.***A**: Cell viability results demonstrate that the addition of hemin augments neuronal damage dose-responsively, with 100 µM hemin chosen as the test concentration (*n* = 3). **B**: Schematic representation illustrates the experimental approach used to simulate SAH in mice and HT22 cells. **C**: Cell viability data reveal that hemin-induced cell damage mimics erastin-triggered cell damage (*n* = 5). **D**: Cell viability data show that hemin-triggered neuronal cell injury is not notably mitigated by inhibitors of autophagy (3‐MA) and caspase-dependent apoptosis (z‐VAD‐fmk), while fer-1 exhibits strong protection against such injury (*n* = 5). **E**: Representative views of Dead/Live staining (red arrows indicate dead cells, white arrows indicate live cells). Scale bar = 100 μm. **F**: Quantitation of MDA level increase in the in vitro SAH model (*n* = 3). **G**: Quantitation of GSH level decrease in the in vitro SAH model (*n* = 3). **H**: Quantitation of intracellular iron increase in the in vitro SAH model (*n* = 3). **I**: Quantitation of increases in ROS accumulation in the in vitro SAH model. **J**: Cell viability shows that si-GPX4 aggravates neuronal death caused by hemin (*n* = 5). **K**: Representative views of Dead/Live staining for increased neuronal death in response to GPX4 knockdown. Scale bar = 100 μm

**Fig. 2:**
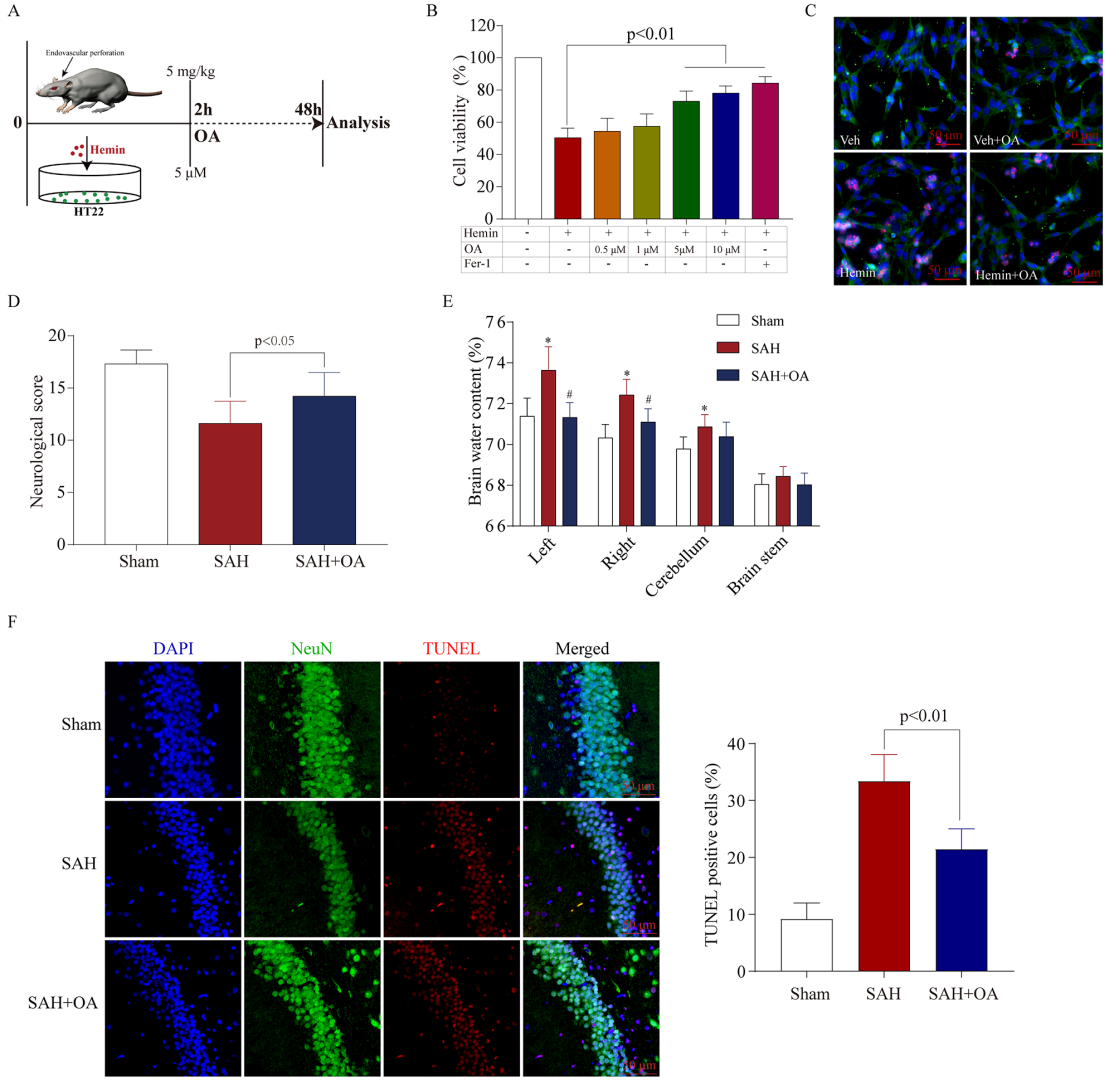
OA alleviates early brain injury and neuronal damage after SAH in vitro and *in vivo.***A**: Schematic representation illustrates the experimental procedure for simulating SAH in mice and HT22 cells. **B**: Cell viability results show that OA addition dose-responsively diminished neuronal damage, with 5 µM OA selected as the test concentration (*n* = 5). **C**: Representative views of Dead/Live staining following OA treatment. Scale bar = 100 μm. **D**: Neurological scores of mice in the three groups following SAH (*n* = 15). **E**: Brain water content comparison among the three groups after SAH (*n* = 5; ^*^p < 0.01 vs. Sham, ^#^*p* < 0.05 vs. SAH; t-test; mean ± SD). **F**: TUNEL assay shows that OA attenuates neuronal death in the hippocampus following SAH. Scale bar = 50 μm

OA (Chengdu MUST Bio-Technology Co., Ltd. Chengdu, China) was dissolved in dimethyl sulfoxide and stored at − 20 °C. To find the best dosage of OA treatment, HT22 cells were subjected to various concentrations, including 0.5 µM, 1 µM, 5 µM, and 10 µM (Fig. 1). In the end, we selected a concentration of 5 µM OA as the best choice for SAH experiments, as shown in Fig. 2b. The Supplementary Fig. S1 displays the structures of the OA. Concentrations of the drug were determined using the previously described method, with certain alterations, including an in vivo dosage of 5 mg/kg [34]. In the context of live SAH, OA was given through the tail vein intravenously at a dosage of 5 mg/kg after 2 h of SAH, which proved advantageous in monitoring post-SAH mortality. To carry out in vitro SAH experiments, cells were seeded into six-well plates and subjected to OA (10 µM) following 2 min of hemin-triggered neuronal damage. Both the sham group and the SAH + vehicle group were administered the same amount of the vehicle at corresponding time points, both in vitro and in vivo.

### Small interfering RNA (siRNA) manipulation in vivo and in vitro

We previously described a protocol [6, 17] that served as the basis for the specific in vivo treatment method. In brief, anesthesia was administered to the animals *via* intraperitoneal injection. The mice were subjected to pentobarbital sodium at a dosage of 50 mg/kg and subsequently secured onto a stereotaxic apparatus manufactured by Narishige (Tokyo, Japan). This positioning allowed for the identification of the bregma location. Following the provided coordinates, a perforation was made in the left hemisphere, positioned 0.2 mm posteriorly, 1 mm laterally, and 2.2 mm below the horizontal plane defined by the bregma. A dedicated syringe from Hamilton(Reno, NV, USA) was employed to inject either GPX4-specific/Nrf2‐specific or scramble siRNA into the lateral ventricle. This injection was administered in a volume of two microliters. In this study, two categories of siRNAs obtained from Thermo Fisher Scientific were employed: one targeted mouse mRNA (Si-GPX4) or Nrf2-specific to inhibit its transcription, while the other was a random siRNA. To enhance the efficiency of silencing, the injection was administered at intervals of 12 and 24 h before SAH. In vitro, the siRNA was incubated in Opti-MEM (Thermo Fisher Scientific) [33]. The siRNA concentrations utilized for HT22 cells were following the manufacturer’s recommended guidelines. Before treatment or collection for message reduction verification, cells were subjected to siRNA treatment for a duration of 12–24 h.

### RNA extraction and quantitative real-time PCR

Total RNA extraction from both cell cultures and hippocampal brain samples was carried out using TRIzol Reagent following the procedural guidelines provided by Gibco, Thermo Fisher Scientific (Waltham, MA, USA). The quantification of RNA was performed using a NanoDrop 2000 spectrophotometer (Thermo Scientific, Bremen, Germany). Afterwards, the RNA underwent reverse transcription. The process of transcription into complementary DNA (cDNA) was accomplished using the RevertAid First Strand cDNA Synthesis Kit (K1622; Thermo Fisher Scientific Inc., Rockford, IL). The determination of mRNA levels in each sample was conducted using qPCR with the SYBR Green Master Mix from Toyobo Co., Ltd. in Osaka, Japan. Normalization of the expression across all samples was achieved by referencing the β-actin gene expression. The qPCR thermocycling regimen commenced with an initial incubation at 45 °C for 2 min, followed by a step at 95 °C for 10 min. Subsequently, a total of 40 cycles were performed, involving denaturation at 95 °C for 15 s, annealing at 60 °C for 1 min, as well as extension at 72 °C for 1 min. The analysis of all samples was conducted three times. PCR amplification was the same as those described previously. Supplementary Table S1 includes the reverse transcription and qRT-PCR.

### Liperfluo staining

After inducing neuronal damage with hemin or erastin, cells were exposed to OA (10 µM) after 2 h. After 48 h, the cells were treated with Liperfluo (10 µM) for 30 min at 37 °C, then harvested using trypsinization, and promptly examined under a fluorescence microscope [35].

### Malondialdehyde (MDA), GSH, and iron assessment

To measure the amount of MDA in HT22 cells, a Lipid peroxidation Assay Kit (Ex/Em 532/553 nm, Ab118970, Abcam, Cambridge, UK) was conducted following the manufacturer’s guidelines [36]. The MDA and iron levels were measured using kits from Jianchen Bioengineer Institute, located in Nanjing, Jiangsu, China, following the manufacturer’s recommended protocols.

### Flow cytometry detection of ROS

After the hemin and drug induction, the HT22 cells were promptly gathered and washed twice with PBS to quantify the accumulation of ROS. The cell suspension was processed and dyed with 10 mM DCFH-DA (Beyotime Biotechnology, Shanghai, China) and then kept at 37 ℃ for 30 min using the ROS Assay Kit following the instructions provided by Beyotime Biotechnology. The proportion of cells undergoing apoptosis in a sample was determined using flow cytometry. The experiments were conducted independently on three separate occasions.

### Ferroxidase assay

Ferroxidase activity quantification was performed using a ferroxidase assay following a protocol previously outlined [16, 17]. In this procedure, the brain sample was introduced into a reaction system comprising PIPES (200 mM, pH 6.5) and apo-TF (50 µM), resulting in a final volume of 200 µL with the specified concentrations. Before incorporation into the assay, ferrous ammonium sulfate (100 µM), functioning as the assay substrate, was dissolved using N2-purged ddH2O. Subsequently, absorbance measurements were monitored using a PowerWave HT microplate spectrophotometer (BioTek, Burlington, VT, USA) under the following conditions: readings at 30-s intervals for 4 min with continuous agitation, at wavelengths of 310 and 460 nm, all maintained at 24 °C. After 4 min, Ferene S (500 µM) was introduced and briefly stirred, followed by an immediate measurement of absorbance (590 nm). During this investigation, which primarily examined enzyme rates, the reactions were under scrutiny for 3 min, characterized by the ferroxidase reaction sustaining a consistent linear phase. Blank reactions were performed using sample buffers containing all components of the reaction mix except for the sample. For transferrin loading (Ferric Gain), the extinction coefficients of Fe were 2.28 mM-1 Fe3 + cm-1, while for Ferrous Loss assays, it was 37.3 mM-1 Fe2 + cm-1.

### Determination of cytokines

Blood specimens were obtained 48 h after surgery/anesthesia. Subsequently, these samples underwent a 15-minute centrifugation at 3,000 rpm before being stored at -80 °C until the testing phase. The concentrations of IL-1β, IL-10, IL-6, and tumor necrosis factor-alpha (TNF-α) in the serum were tested *via* enzyme-linked immunosorbent assay (ELISA) kits provided by R&D Systems (USA).

### Cell viability detection

The assessment of viability of cells was carried out using the WST-1 technique and a kit following the guidelines provided by Jianchen Bioengineer Institute (Nanjing, Jiangsu, China). The viability assessment for the HT22 cells was conducted 48 h after the initiation of the hemin treatment. Then, the medium was replaced with fresh medium containing 10 µl WST-1 reagent. Relative cell numbers were calculated by measuring the absorbance of each well at 450 nm.

### Dead/Live assay for HT22 cells

A previously outlined protocol of a Dead/Live assay [17] was utilized for HT22 cell analysis. The HT22 cells were subjected to two washes and subsequent suspension in PBS. PI (4 µM), along with calcein-AM (3 µM), was introduced to the samples, which were then kept in a light-restricted environment at ambient temperature for 20 min. Following the PBS rinsing and suspension of cells, we subjected them to examination using a Leica fluorescence microscope system (DMIL 4000B). The distinction between dead and live cells was made based on the red fluorescence emitted as a result of bound PI for dead cells and the green fluorescence generated by calcein-AM for live cells. To determine cell viability, we computed the percentage in relation to the value observed in the control cultures. This experimental procedure was conducted independently in triplicate.

### TUNEL assay

The assessment of neuronal death in the brain cortex involved the application of a TUNEL assay, following a protocol as detailed in previous references [6, 17]. In brief, sections of mouse brain samples were subjected to a paraformaldehyde solution (4%) for 30 min at ambient temperature. Subsequent to this step, the sections were immersed in a 0.2% H_2_O_2_-containing methanol solution for another 30 min. Then, each sample received 50 µL of TUNEL reaction mixture, followed by a light-restricted incubation with humidity for 1 h at 37 °C. Afterward, the slides were subjected to a light-restricted staining process with DAPI for a duration of 5 min at ambient temperature to label the cell nuclei. Following this step, the nucleus visualization was achieved using Nikon laser confocal microscopy (A1, Tokyo, Japan).

### Double immunofluorescence staining

Cell and neuronal counting were conducted through double immunofluorescence staining of GPX4, a protein associated with ferroptosis, and NeuN, a marker specific to neuronal staining. Detailed procedures for this method have been extensively described in our previous studies [17, 31]. In short, following a 24-hour brain fixation with a 4% solution of formaldehyde at 4 °C, the brains underwent dehydration in a solution of 30% sucrose. The brain specimens were sliced into sections, each 10 μm thick, and were used for further analysis. These sections were subjected to an overnight incubation at 4 °C with such primary antibodies (all from Abcam, except for anti-GPX4 from Affinity) as rabbit anti-NeuN polyclonal antibody (pAb; dilution 1:200, ab128886), rabbit anti-GPX4 monoclonal antibody (mAb; 1:200, ab125066), rabbit anti-AIFM2/FSP1 antibody (dilution 1:1000, DF8636), and rabbit anti-Iba1 (mAb; dilution 1:100, ab178847). Next, the brain sections underwent three rounds of PBS washing and were subsequently treated with a goat pAb secondary antibody (Abcam) targeting rabbit IgG - H&L (dilution 1:200, goat pAb, ab150077) for a duration of 2 h at ambient temperature. Next, the sections underwent an incubation with DAPI (4’,6-diamidino-2-phenylindole) for 5 min. They were then examined and analyzed using Nikon laser confocal microscopy.

### Western blot analysis

The Western blot assays were conducted following the methods described earlier [17, 31]. Protein extraction was performed on the HT22 sample. Next, 50 mg of protein lysates underwent separation through sodium dodecyl sulfate-polyacrylamide gel electrophoresis. Following this, proteins that were separated were transferred onto Immobilon nitrocellulose membranes provided by Millipore (Boston, MA, USA). These membranes underwent a blocking process in 5% skim milk-containing TBS-T, followed by a 1-h incubation with Tween 20 (0.1%) at ambient temperature. Afterward, the membranes were subjected to overnight incubation at 4 °C with the specified primary antibodies: anti-GPX4 (mAb; dilution 1:1000, rabbit mAb, ab125066; Abcam), anti-Nrf2 (dilution 1:1000, rabbit pAb, ab92946; Abcam), rabbit anti-AIFM2/FSP1 (dilution 1:1000, Affinity, DF8636), rabbit anti-Heme Oxygenase 1 (dilution 1:2000, rabbit pAb, ab13243; Abcam), anti-FTH1 (dilution 1:1000, rabbit mAb, ab18878; Abcam), rabbit anti-CD206 (dilution 1:200, DF4149, Affinity), rabbit anti-CD32 (dilution 1:500, DF6402, Affinity), anti-SLC7A11 (1:2000, rabbit mAb, ab175186; Abcam), anti-SLC3A2 (rat pAb, dilution 1:1000, #PA5-96401, Thermo Fisher Scientific), anti-NQO1 (dilution 1:2000, rabbit pAb, ab34173; Abcam), anti-GAPDH (dilution 1:1000, mouse mAb, ab8245; Abcam). Following three TBST washes, membranes were exposed to HRP-conjugated secondary antibodies (dilution 1:5000) specific to rabbit or mouse IgG. The incubation occurred at ambient temperature for 1.5 h. The protein band visualization and quantification were carried out utilizing a Bio-Rad imaging system (Hercules, CA, USA) and the ImageJ method.

### Statistical analysis

Each group was subjected to testing in over three replicate experiments, and the findings were depicted as the mean values along with their respective standard deviations (SDs). Statistical assessments were executed utilizing IBM SPSS version 19 (Armonk, NY, USA). When comparing two groups, the Student’s t-test was employed, while in the case of comparing two independent variables, a one-way analysis of variance (ANOVA) was carried out, followed by a subsequent Bonferroni *post-hoc* test. For the analysis of data that did not conform to normal distribution or exhibited nonhomogeneous variance, the Kruskal‒Wallis test was applied, followed by a subsequent Dunn’s *post-hoc* test. Data were deemed significant at a P-value of less than 0.05.

## Results

### Ferroptosis assumes a critical role in SAH and hemin-induced neuronal injury in vitro

Extensive research has examined cell death mechanisms in neuronal cells by utilizing hemoglobin or hemin within controlled laboratory conditions [13]. Notably, the evaluation of HT22 cytotoxicity induced by hemin at 100 µmol/L was visually represented in Fig. 1A and B. Intriguingly, the in vitro impacts of hemin-induced neuronal damage closely mimicked those observed in erastin-triggered ferroptosis (Fig. 1C). In addition, it was noted that the protection against hemin-induced neuronal injury in vitro remained unaffected by z-VAD-fmk and 3-MA without GPX4 knockdown, which were inhibitors targeting caspase-dependent apoptosis and autophagy. In contrast, the administration of ferrostatin-1 (Fer-1), a chemical inhibitor specifically designed to counteract ferroptosis, exhibited robust protective properties against hemin-triggered neuronal injury (Fig. 1D and E). Following hemin-induced HT22 damage, there was a significant increase in intracellular MDA (Fig. 1F), iron (Fig. 1H), and ROS (Fig. 1I) levels, while intracellular reductive GSH (Fig. 1G) levels showed a significant decrease. 12 h before the addition of hemin, either GPX4-specific or random siRNA was administered. Lower GPX4 expression led to more neuronal death (Fig. 1J and K). In vitro, these studies demonstrate that ferroptosis plays a significant role in both SAH and hemin-induced neuronal damage. The induction of GPX4 is a component of the adaptive response in neurons to ferroptosis, reducing GPX4 levels beyond those that promote ferroptosis following SAH.

### OA alleviates EBI and neuronal damage after SAH in vitro and in vivo

To elucidate the neuroprotective effects of OA, we employed both hemin-induced and endovascular perforation techniques to establish an SAH model in both live animals and laboratory settings (as shown in Fig. 2A). Neuronal damage caused by hemin was effectively diminished by the addition of OA in a dose-responsive manner, as observed in Fig. 2B and C. Furthermore, the neuroprotective impact of OA on neurons following SAH was comparable to Fer-1’s ability to inhibit ferroptosis, as shown in Fig. 2B. To further confirm the potential of OA in reducing initial brain damage in a mouse model of SAH. The OA administration before SAH modeling improved neurological impairments (Fig. 2D) and early brain swelling (Fig. 2E). Additionally, TUNEL immunocytochemistry staining demonstrated that OA effectively decreased neuronal death in comparison to the SAH group (Fig. 2F). The data points to the potential of OA in diminishing neuronal death and ameliorating EBI following SAH.

### OA against ferroptosis after SAH in vivo

**Fig. 3:**
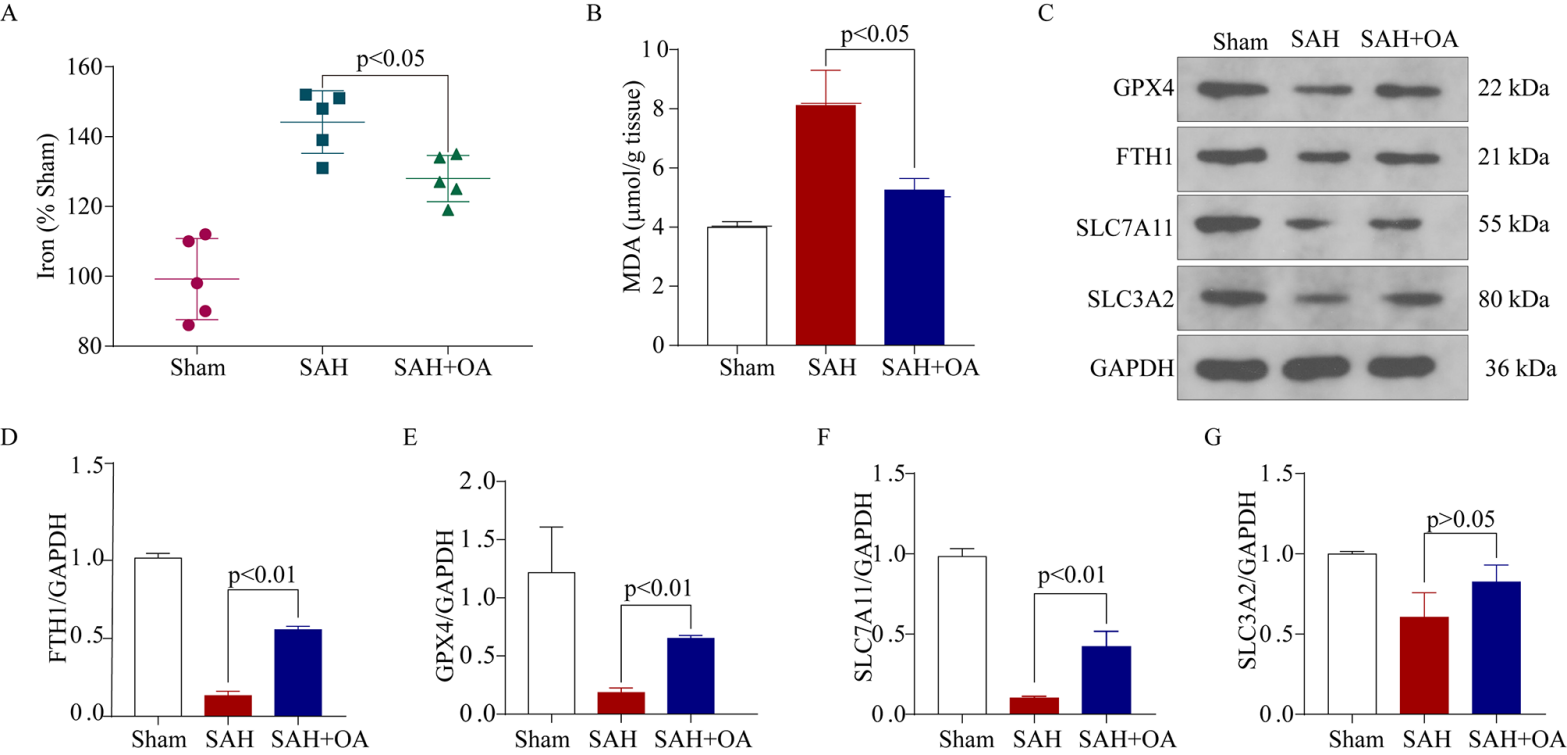
OA alleviates EBI and neuronal damage after SAH *in vivo.***A**: Hippocampal iron levels in the three groups decreased after OA treatment (*n* = 5). **B**: Hippocampal MDA levels in the three groups decreased after OA treatment (*n* = 3). **C**: Western blot illustrated the levels of ferroptosis-associated proteins, including GPX4, FTH1, SLC7A11, and SLC3A2, in the brain cortex after SAH. **D**: Quantification of FTH1 level increase in response to OA treatment after SAH (*n* = 3). **E**: Quantification of GPX4 level increase in response to OA treatment after SAH (*n* = 3). **F**: Quantification of SLC7A11 level increase in response to OA treatment after SAH (*n* = 3). **G**: Quantification of SLC3A2, with no significant difference observed among the three groups (*n* = 3)

To investigate if OA can relieve neuronal ferroptosis in both living organisms and laboratory settings, we conducted an in vivo ferroxidase assay. After SAH, the findings indicated an increase in iron levels in the hemisphere after 48 h, and the presence of OA prevented the buildup of iron (as depicted in Fig. 3A). Following SAH, the levels of MDA in the tissue were elevated, but they were significantly reduced after treatment with OA (as shown in Fig. 3B).In SAH mice, we also assessed the protein levels associated with ferroptosis using Western blot (Fig. 3C). Following SAH, the levels of FTH1, GPX4, and SLC7A11 exhibited a decline (Fig. 3D-F), which was subsequently reversed by OA treatment. SLC3A2 levels did not show any notable disparity between the SAH and SAH + OA groups, as depicted in Fig. 3G.

### OA against ferroptosis after SAH in vitro

**Fig. 4:**
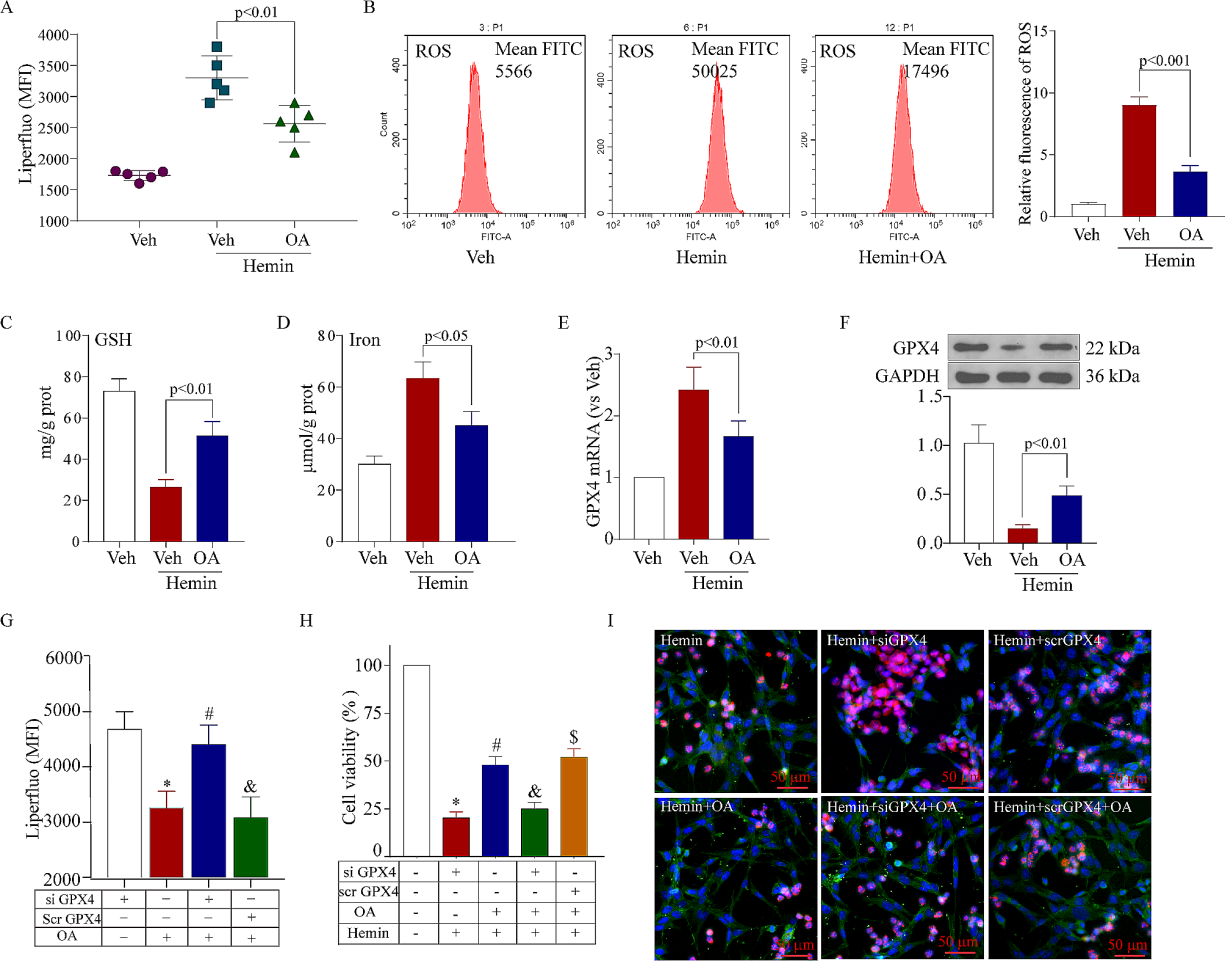
OA against ferroptosis after SAH in vitro, **A**: Liperfluo shows that OA alleviates intracellular lipid peroxidation after SAH in vitro (*n* = 5). **B**: Flow cytometry shows that OA decreased the intracellular ROS levels after SAH in vitro. **C**: OA increased the levels of GSH in the in vitro SAH model (*n* = 3), **D**: OA increased the levels of intracellular iron in the in vitro SAH model (*n* = 3), **E**: RT-qPCR shows that OA increased the mRNA expression of GPX4 in an in vitro SAH model (*n* = 3), **F**: Western blot shows that OA facilitated the protein expression of GPX4 in an in vitro SAH model. **G**: Liperfluo showed that OA alleviated intracellular lipid peroxidation, which was reversed after GPX4 knockdown (*n* = 5, ^*^p < 0.001 vs. hemin + si-GPX4 group, ^#^*p* < 0.001 vs. hemin + OA group, ^&^*p* < 0.001 vs. hemin + OA + si-GPX4 group), **H**: Cell viability shows that OA alleviated cell viability after SAH, which was reversed after GPX4 knockdown (*n* = 5, ^*^p < 0.001 vs. hemin + si-GPX4 group, ^#^*p* < 0.001 vs. hemin + OA group, ^&^*p* < 0.001 vs. hemin + OA + si-GPX4 group). **I**: Representative dead/live staining shows that OA alleviated cell death after SAH but that this effect was reversed after GPX4 knockdown. Scale bar = 100 μm

To determine if OA could prevent hemin-induced ferroptosis of neurons in vitro and if GPX4 played a role in its regulation, liperfluo staining, flow cytometry, and relevant assay kits were used to evaluate lipid peroxidation, ROS, GSH, and iron levels. The findings indicated that OA treatment inhibited lipid peroxidation (Fig. 4A), ROS (Fig. 4B), and iron (Fig. 4C), while simultaneously enhancing the levels of GSH (Fig. 4D). The GPX4 mRNA and protein expression was escalated by RT-qPCR (Fig. 4E) and Western blot (Fig. 4F). The findings suggested the neuroprotective effects of OA in curbing the ferroptosis of neurons. To delve deeper into the subject, we experimented to examine if reducing the levels of GPX4 in neurons could diminish the neuroprotective effects of OA through ferroptosis following cell death induced by hemin. 12 h before the addition of hemin, either GPX4-specific or random siRNA was administered. After GPX4 knockdown, the findings indicated that lipid peroxidation (Fig. 4G) and neuronal cell death (Fig. 4H and I) were exacerbated, consequently reversing the neuroprotective effects of OA. Therefore, these findings validated that the preservation of nerve cells in OA might rely on ferroptosis, and the activation of GPX4 was crucial for safeguarding against hemin-induced ferroptosis in OA.

### OA treatment inhibited microglial activation and ameliorated neuroinflammation following SAH

**Fig. 5:**
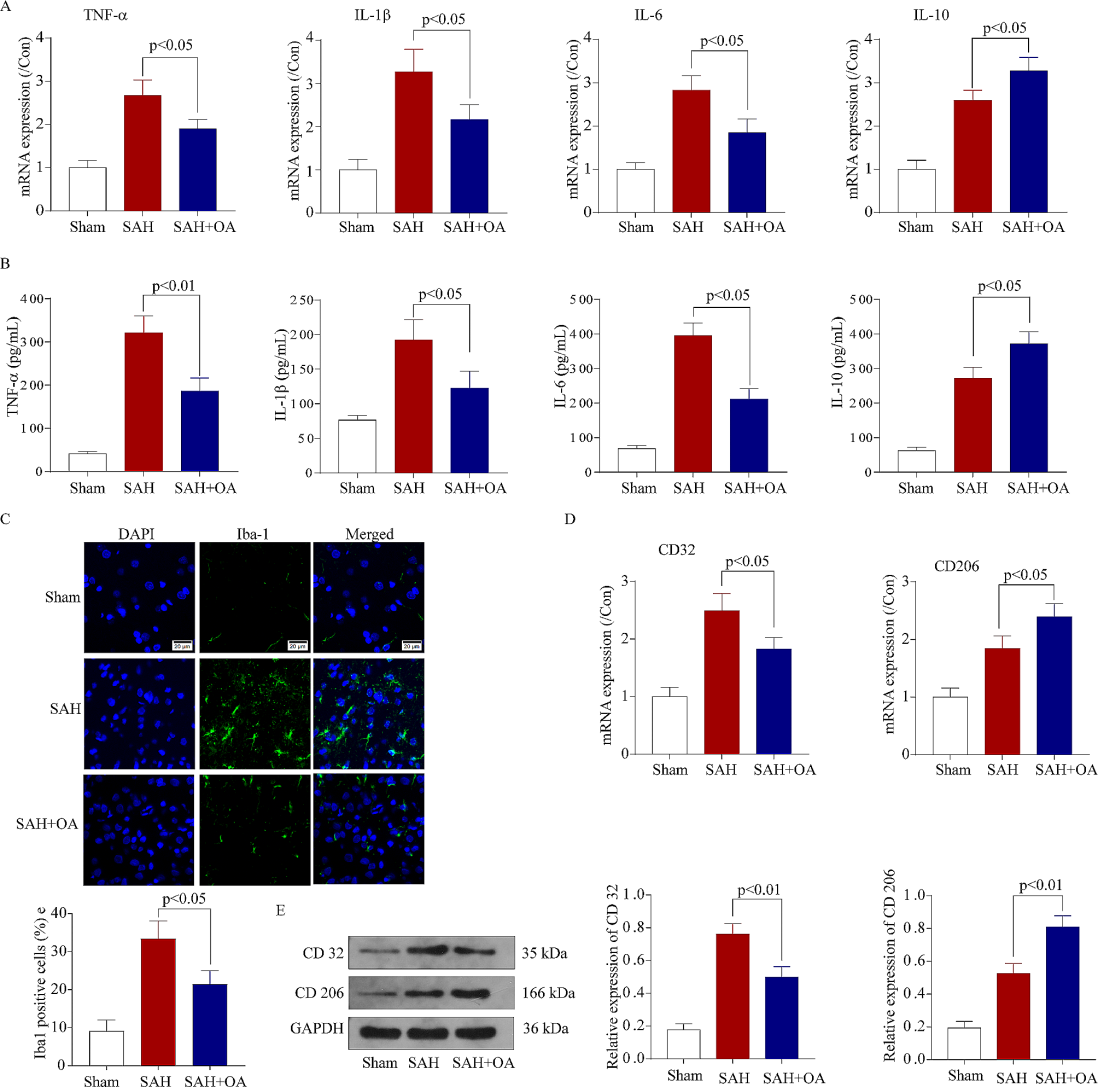
OA treatment inhibited microglial activation and ameliorated neuroinflammation following SAH. **A**: qPCR assay indicating the mRNA expression levels of TNF-a, IL-6, IL-1β, and IL-10 in hippocampal tissues following SAH **B**: ELISA analysis indicates the protein secretion levels of TNF-a, IL-6, IL-1β, and IL-10 in hippocampal tissues following SAH. **C**: Immunofluorescence staining for Iba-1 in hippocampal tissues following surgery/anesthesia. **D**: qPCR assay indicating the mRNA expression levels of CD-32 and CD-206 in hippocampal tissues following SAH. **E**: Representative Western blot bands (as normalized to GAPDH) illustrate the protein levels of CD 32 and CD206 in hippocampal tissues following SAH. The data are summarized as mean ± SD (*n* = 3). Scale bar = 20 μm

The development of neuroinflammation relies heavily on the activation of microglia and the brain damage caused by SAH. This study proceeded to explore the possible impact of OA on microglial activation and the dynamic interplay between M1 and M2 phenotypes. The qPCR assay results demonstrated a substantial reduction in the mRNA levels of proinflammatory cytokines, including IL-1β, IL-6, and TNF-α, and chemokines (Fig. 5A), coupled with a notable escalation in the mRNA levels of the anti-inflammatory cytokine IL-10 (Fig. 5A). Moreover, the ELISA results on the concentrations of proinflammatory factors in brain tissue were in line with the mRNA expression findings (Fig. 5B).To examine the Iba-1 expression, the immunofluorescence assay was conducted, providing additional evidence for the aforementioned findings (Fig. 5C).In the end, qPCR tests and Western blot were conducted to assess the degrees of microglial M1/M2 polarization, specifically examining CD32 (referred to as M1 polarization) and CD206 (referred to as M2 polarization). The qPCR assay results demonstrated that OA significantly reduced the mRNA levels of CD32 (Fig. 5D) while increasing the mRNA levels of CD206 (Fig. 5D). The Western blot findings were in agreement with the mRNA expression outcomes (Fig. 5E). Hence, these findings indicated that OA can promote M2 phenotypic polarization in microglia and alleviate neuroinflammation following SAH.

### OA as a neuroprotection involving the transcriptional regulator Nrf2

**Fig. 6:**
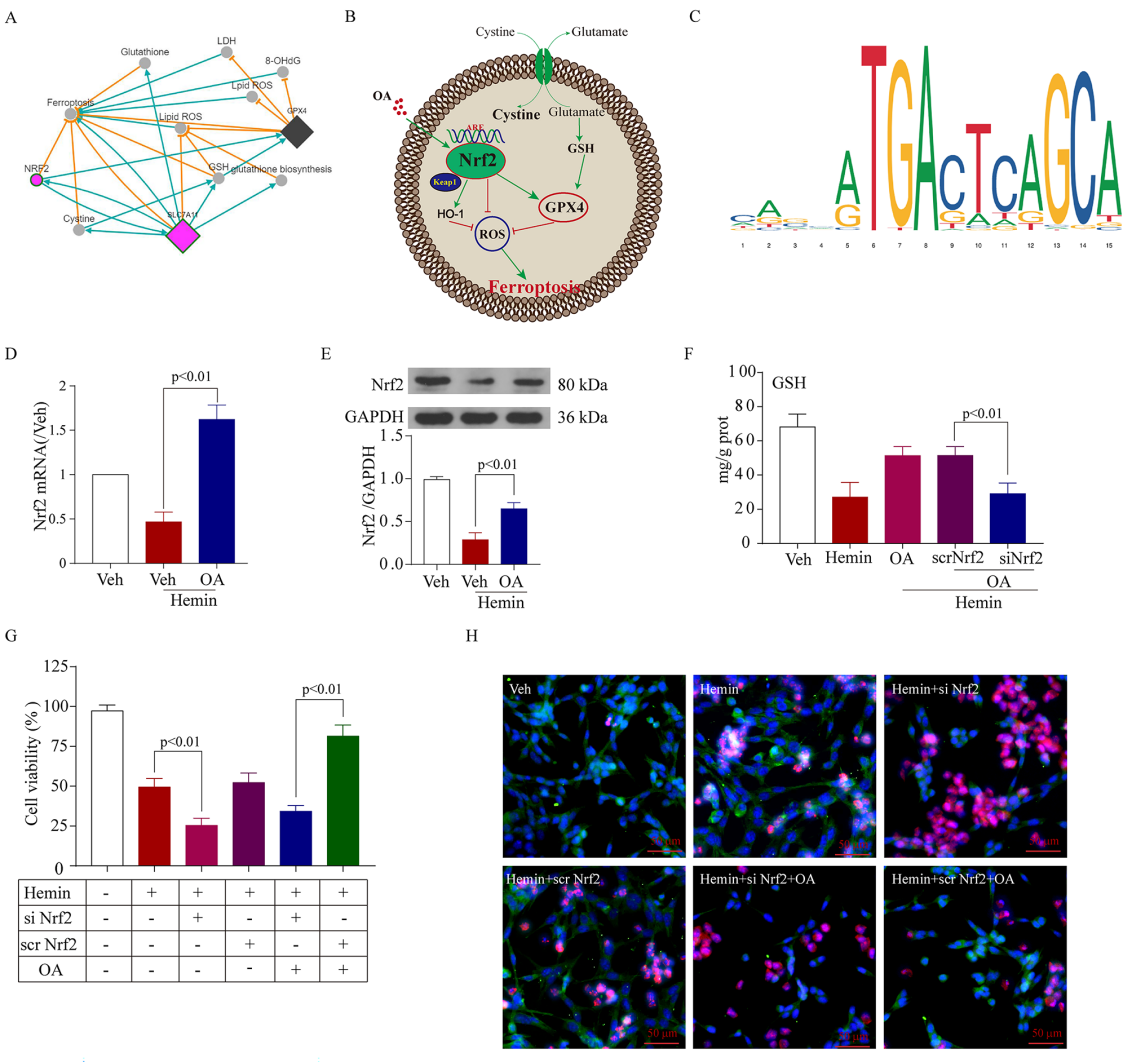
OA as a neuroprotection involving the transcriptional regulator Nrf2. **A**: FerrDb V2 (accessible at http://www.zhounan.org/ferrdb/current/) suggests the relationship between ferroptosis and Nrf2. **B**: An illustrative diagram of the proposed model elucidating the mechanisms of Nrf2-mediated regulation of ferroptosis in EBI following SAH. **C**: Prediction of GPX4 promoter using the NCBI database (accessible at https://www.ncbi.nlm.nih.gov/) and JASPAR database (accessible at https://jaspar.genereg.net/). **D**: RT-qPCR results reveal that OA elevates the expression of Nrf2 mRNA in the in vitro SAH model (*n* = 5). **E**: Western blot data unveil that OA increases the expression of Nrf2 protein in in vitro SAH (*n* = 3). **F**: si-Nrf2 partially prevented the OA-induced increase in GSH in in vitro SAH (*n* = 5). **G**: Knockdown of Nrf2 partially prevented the OA-induced increase in cell viability in in vitro SAH (*n* = 5). **H**: Representative views of Dead/Live staining show that OA alleviates cell death after SAH but that this effect was reversed after Nrf2 knockdown. Scale bar = 100 μm

The aforementioned findings indicated that OA can mitigate initial brain trauma and neuronal harm following SAH both in laboratory settings and in living organisms by suppressing ferroptosis. Nevertheless, the possible route and mechanism remain uncertain. After conducting a thorough examination of the relevant literature and FerrDb V2 (http//www.zhounan.org/ferrdb/current/), we discovered a correlation between ferroptosis and Nrf2 (as depicted in Fig. 6A). The binding site ARE (antioxidant response element) [37] is used by Nrf2, a crucial factor for regulating the expression of more than 250 genes as a transcriptional regulator. The Nrf2-Keap1 protein complex assumes a pivotal role in curtailing ferroptosis by orchestrating the cellular antioxidant response and repressing electrophilic and oxidative stress, maintaining proteostasis, managing xenobiotic/drug metabolism, controlling iron/heme metabolism, and regulating carbohydrate and lipid metabolism [38]. After carotid endarterectomy, the expression of Nrf2, SOD, CAT, and GPX can be enhanced by OA as well [39]. As a result, we postulated that the activation and excessive expression of Nrf2 also boosted the transcription of genes related to anti-ferroptosis and antioxidative stress following treatment with OA (Fig. 6B). According to the information available in the NCBI database (accessible at https//www.ncbi.nlm.nih.gov/) and JASPAR database (https://jaspar.genereg.net/), it was also predicted that there were Nrf2 binding sites in the GPX4 promoter (Fig. 6C). In the in vitro SAH model experiments, it was noted that the mRNA (Fig. 6D) and protein (Fig. 6E) levels of Nrf2 exhibited an increase following OA treatment.

As the regulatory functions of Nrf2 were strong, including modulating the cellular antioxidant response and oxidative stress, the observation of increased Nrf2 in vivo after OA treatment did not indicate whether overexpression of Nrf2 contributed to preventing ferroptosis or was just the result of it. To ascertain if Nrf2 controls transcription and inhibits ferroptosis in neurons, we employed small interfering RNAs (siRNAs) to decrease the levels of Nrf2. The results also revealed that neuronal ferroptosis was aggravated after Nrf2 knockdown and weakened the neuroprotection of OA (Fig. 6F and G, and 6H).

### Knockout of Nrf2 promotes ferroptosis and reverses the neuroprotection of OA after SAH

**Fig. 7:**
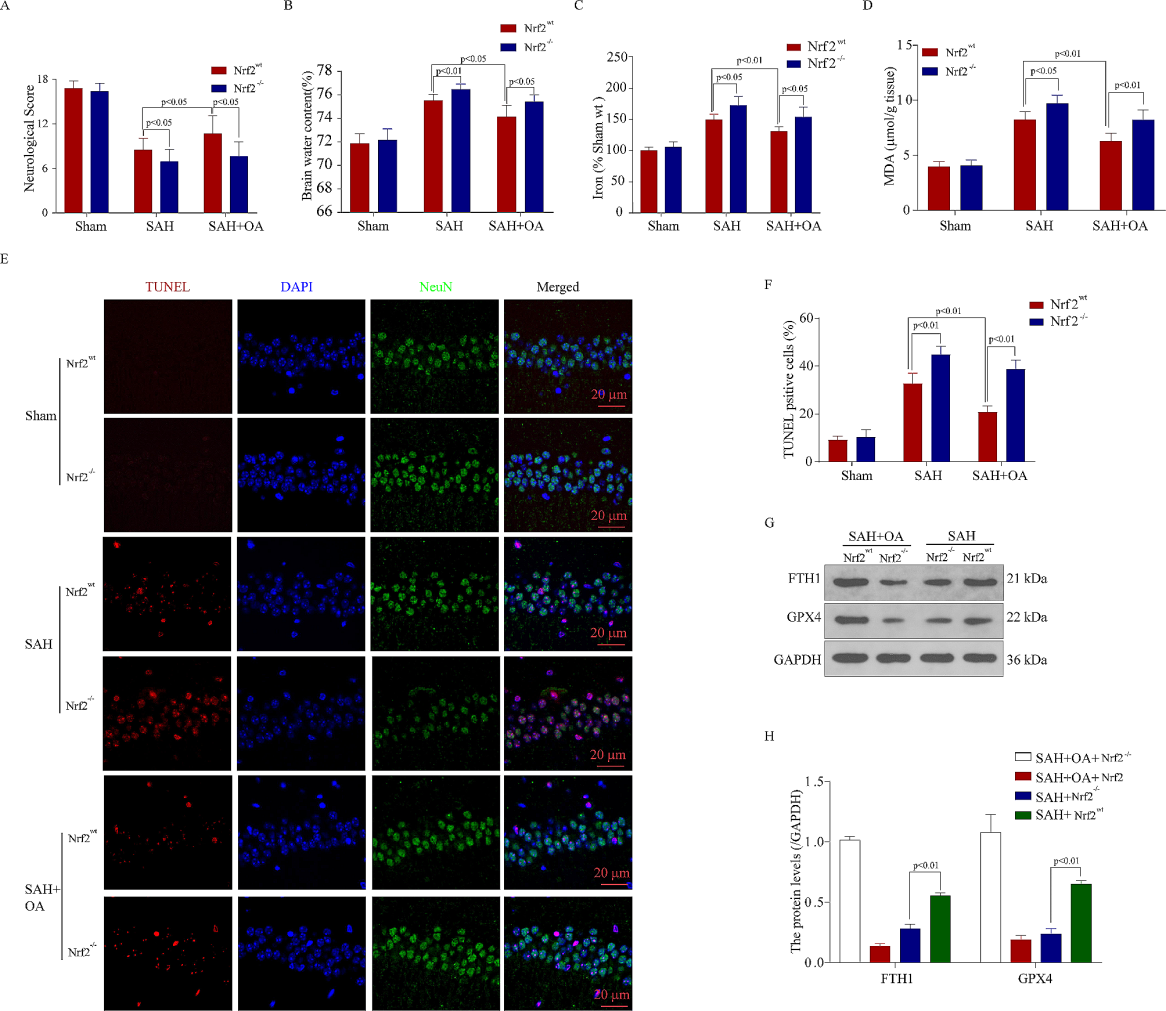
Knockout of Nrf2 promotes ferroptosis and reverses the neuroprotection of OA after SAH. **A**: Neurological scores of Nrf2^−/−^ and Nrf2^wt^ mice in the three cohorts following SAH (*n* = 10, t-test, mean ± SD). **B**: Brain water content of Nrf2^−/−^ and Nrf2^wt^ mice in the three groups after SAH (*n* = 5). **C**: Hippocampal iron levels of Nrf2^−/−^ and Nrf2^wt^ mice in the three groups after SAH (*n* = 5). **D**: Hippocampal MDA levels of Nrf2^−/−^ and Nrf2^wt^ mice in the three groups after SAH (*n* = 5). **E**: TUNEL staining shows that OA attenuates neuronal death, and Nrf2 knockout partially prevented the OA-induced decrease in cell death in the hippocampus following SAH. Scale bar = 50 μm. **F**: show that knockout of Nrf2 further increased neuronal apoptosis after SAH (*n* = 5). **G**: Western blot illustrated the expression levels of GPX4 and FTH1 in the Nrf2^−/−^ and Nrf2^wt^ mouse brain cortex after SAH. **H**: Quantification of GPX4 and FTH1. Knockout of Nrf2 reversed the OA-induced increase in GPX4 and FTH1 protein levels in the hippocampus following SAH (*n* = 3)

To address whether the same results occurred in vivo after SAH, we used Nrf2 knock-out (Nrf2^−/−^) and Nrf2 wild-type (Nrf2wt) mice. Figure 2A shows the utilization of identical experimental conditions and intervention factors. As anticipated, the Nrf2 knockout (Nrf2^−/−^) group exhibited augmented neurological impairments (Fig. 7A) and initial brain swelling (Fig. 7B), while the ferroxidase examination (Fig. 7C) indicated a notable rise in iron buildup and MDA concentrations (Fig. 7D) in Nrf2^−/−^ mice when compared to Nrf2wt mice following SAH. In Nrf2^−/−^ mice after SAH, TUNEL staining (Fig. 7E and F) revealed an increased amount of neuronal damage. Additionally, the anti-ferroptosis function of OA was weakened in Nrf2^−/−^ mice. Additionally, we observed the protein levels of regulators associated with ferroptosis and demonstrated the reduction of GPX4 in Nrf2^−/−^ mice following SAH (Fig. 7G and H). The experiment also indicated that OA alone had a weak effect on GPX4 without Nrf2 synergy.

### Knockout of Nrf2 reversed the neuroprotection and revoked the anti-neuroinflammation of OA

**Fig. 8:**
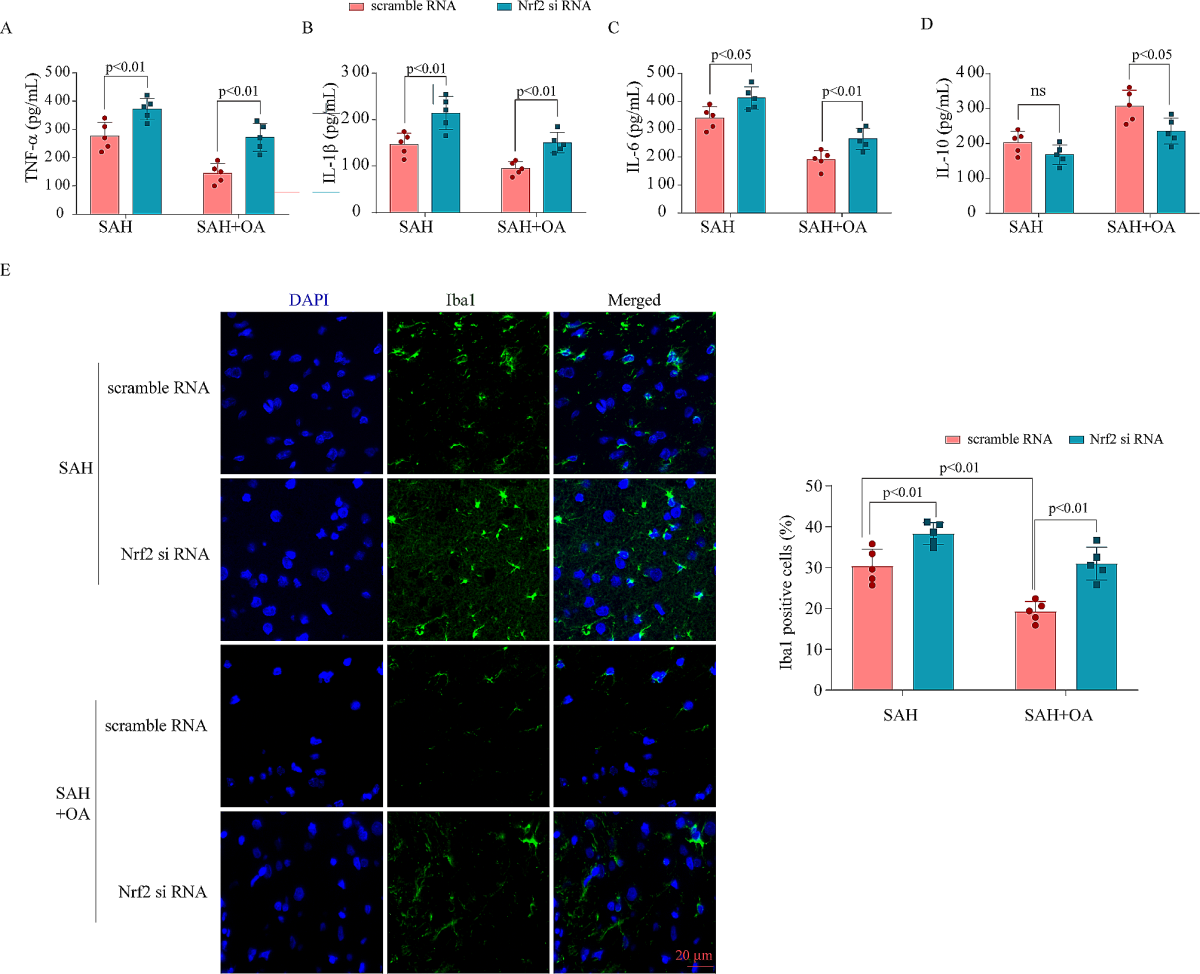
Knockout of Nrf2 reversed the neuroprotection and revoked the anti-neuroinflammation of OA. **A-D**: ELISA analysis indicated the protein secretion levels of TNF-a, IL-6, IL-1β, and IL-10 in hippocampal tissues after Nrf2 was knocked down following sah. **E**: Immunofluorescence staining for Iba-1 in hippocampal tissues after Nrf2 knockdown following SAH

Additionally, we measured the levels of proinflammatory factors in brain tissue following the knockout of the Nrf2 gene during SAH. After Nrf2 gene knockout, the ELISA findings indicated a notable escalation in the concentrations of IL-6, IL-1β, and TNF-α, while IL-10 exhibited a notable decrease (Fig. 8A and D). To investigate the expression of the microglial marker Iba-1 (Fig. 8E), the immunofluorescence assay further confirmed the aforementioned findings. Therefore, these findings suggest that OA has the potential to relieve EBI after SAH by suppressing neuroinflammation through the Nrf2 signaling pathway.

### OA drives the transcriptional regulator Nrf2 to upregulate GPX4 and FSP1

**Fig. 9:**
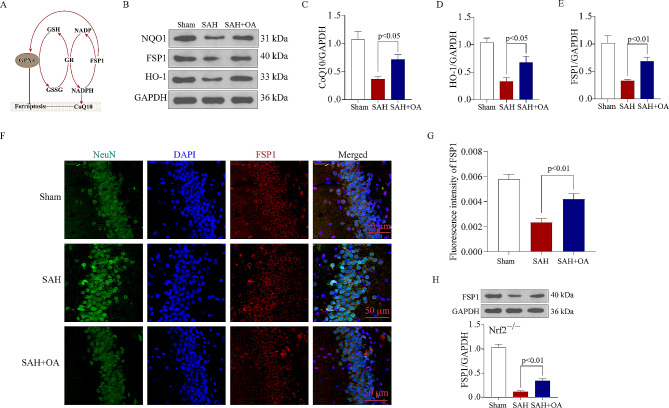
OA drives the transcriptional regulator Nrf2 to upregulate GPX4 and FSP1. A: An illustrative diagram of the proposed model delineating the mechanisms of FSP1-mediated regulation of ferroptosis in EBI following SAH. B: Western blot data illustrate the levels of NQO1, FSP1, and HO-1 in the brain cortex after SAH. C: Quantification of CoQ10 among the three groups. OA increased the expression of CoQ10 (*n* = 3). D: Quantification of HO-1 among the three groups. OA increased the expression of HO-1 (*n* = 3). E: Quantification of FSP1 among the three groups. OA increased the expression of FSP1 (*n* = 3). F: Representative views of immunofluorescence staining of FSP1. Scale bar = 50 μm. G: Quantitative analysis of the of FSP1 (*n* = 3). H: Western blot data show that OA fosters the protein levels of FSP1, and Nrf2 knockout does not prevent the OA-induced escalation in GPX4 levels after SAH (*n* = 3)

The study observations suggest that OA can trigger a transcriptional reaction through Nrf2, leading to the upregulation of GPX4. This upregulation of GPX4 plays a crucial role in preventing ferroptosis. Whether other pathways are regulated by OA that can induce ferroptosis is unclear. NADPH is essential to balance lipid hydroperoxides. According to Kleszczynski [40], the gene expression of heme oxygenase-1 (HO-1) and NADPH is increased by OA. Additionally, our prior investigation demonstrated the significant involvement of FSP1 in acute diseases of the CNS [15]. We tested whether OA could also activate the FSP1 pathway and prevent ferroptosis (Fig. 9A). The findings demonstrated a notable rise in the levels of FSP1, NQO1, and HO-1 following OA treatment in SAH mice, as depicted in Fig. 9B and E. The results of immunocytochemistry indicated a reduction in the intensity of the FSP1 signal (red) following SAH, while OA enhanced the intensity of FSP1 in hippocampal neurons (Fig. 9F and G). To assess if Nrf2 also controlled FSP1, we examined the levels of FSP1 expression in mice lacking Nrf2 following SAH. The Western blot findings demonstrated that the level of FSP1 was still elevated in Nrf2^−/−^ mice after SAH, even with OA treatment (Fig. 9H). Together, these unexpected findings indicate that OA may directly activate FSP1 and that the transcription factor Nrf2 is likely not responsible for its regulation. Nevertheless, the complete comprehension of the molecular mechanisms governing this twofold method of transcriptional control remains elusive, necessitating further experimental validation.

## Discussion

**Fig. 10:**
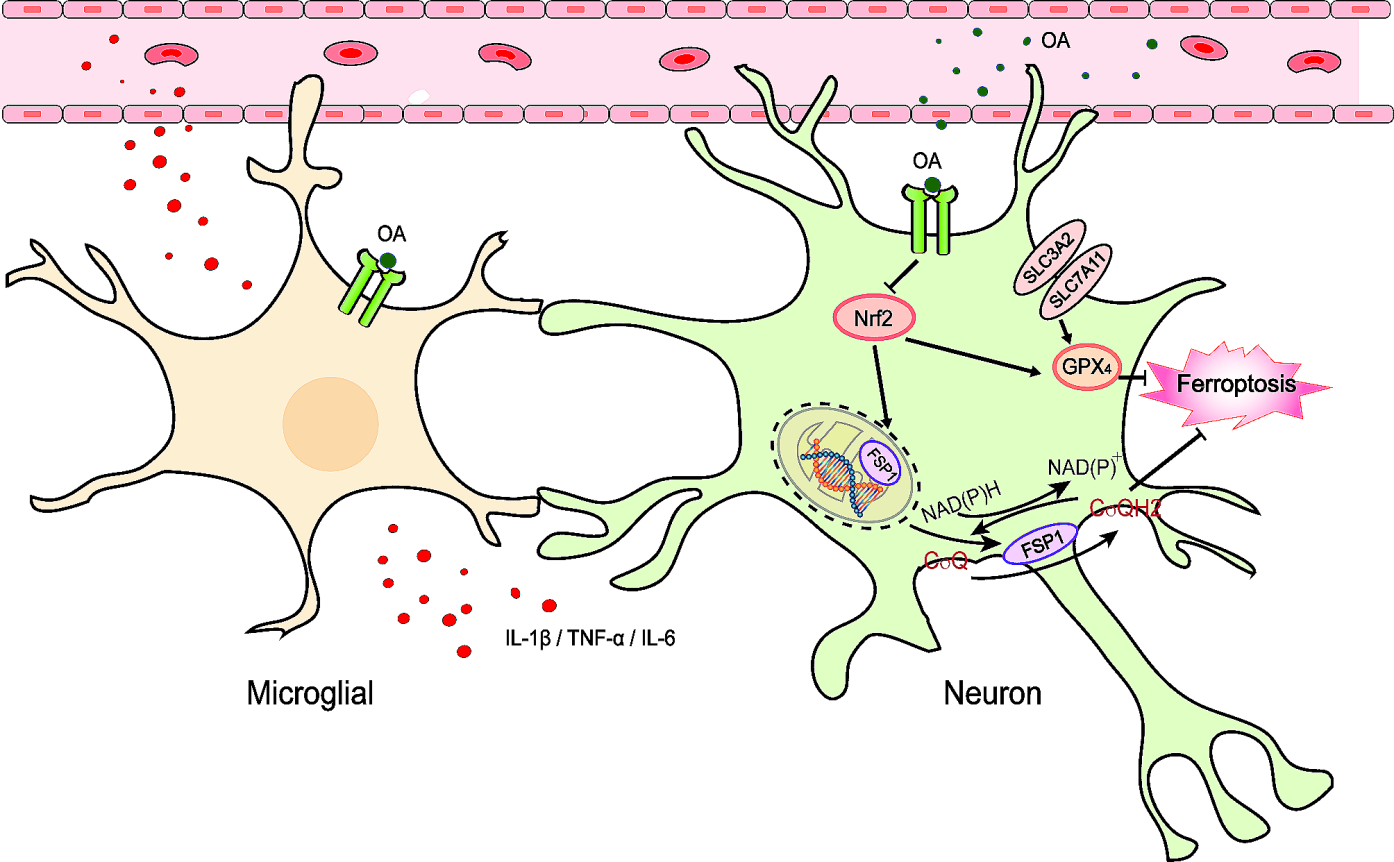
A hypothesized illustration of possible mechanisms through which might exert its regulatory influence on ferroptosis following SAH through the Nrf2/GPX4 and CoQ10-FSP1 pathways

The complex molecular processes responsible for EBI following SAH have been extensively studied, and the majority of failures in clinical drug research and development have been demonstrated through randomized control trials focusing on anti-apoptosis, anti-CVS, and autophagy [4, 5, 41]. Up until now, there has been no effective drug or treatment capable of delaying or averting the death of nerve cells after SAH. Based on a substantial amount of existing evidence and our previous research, it appears that ferroptosis holds potential as a viable target [15]. This research delved into the potential therapeutic benefits of OA for addressing EBI using in vivo and in vitro SAH models. We have identified the suppressive effects of OA on neuronal ferroptosis following SAH through the modulation of the Nrf2/GPX4 and FSP1/CoQ10 pathways (Fig. 10). The results highlight that (i) ferroptosis confers a vital role in both in vivo SAH models and in vitro neuronal damage triggered by hemin; (ii) OA diminishes neuronal damage by impeding ferroptosis and neuroinflammation in both in vitro and in vivo SAH models; (iii) the neuroprotective properties of OA are mediated through the Nrf2/GPX4 pathway, and the inhibitory effects of OA on ferroptosis and neuroinflammation depend on the transcriptional response of Nrf2; (iv) silencing Nrf2 using siRNA transfection does not exhibit detrimental effects on neurons; and (v) the FSP1-CoQ10 pathway may render substantial and parallel effects on preventing ferroptosis during EBI after SAH, working in conjunction with GPX4.

The complexity of the pathological process following SAH is highly intricate. Numerous prior investigations, including our research, have showcased the possible molecular pathways that could be pivotal in the context of EBI after SAH. Even though, several medications have been formulated and elucidated using animal models to shed light on this complex condition. Nevertheless, there is no evidence of the effectiveness of any of these medications in real-life patients [4, 5]. Moreover, in the ongoing examination, we discovered that z-VAD-fmk and 3-MA, which both hinder cell death and self-degradation, were unable to completely mitigate the neuronal harm caused by hemin in a laboratory setting. In the in vitro SAH model, we discovered that ferrostatin-1, a ferroptosis inhibitor, effectively decreases neuronal death.

Considerable investigation has been undertaken regarding ferroptosis, a newly identified mechanism of programmed cell death [19]. This unique form of cell death is distinguished by its iron-dependent nature and is of significant importance in the context of neuronal death in the acute CNS setting [17]. Alim [33] found that the use of selenium supplements in pharmacology effectively inhibits GPX4-dependent ferroptosis and cell death induced by ER stress or excitotoxicity. Additionally, it has the potential to reduce cell death and enhance cellular activity after ischemic or hemorrhagic stroke. Ferroptosis inhibitors, such as liproxstatins, iron chelators, and ferrostatins, have demonstrated a significant reduction in EBI and neuronal cell death resulting from ischemic injury during ischemic stroke [33, 42–44]. Tuo [44] provided evidence and backing for the effectiveness of ferroptosis inhibitors and specific selenium compounds in mitigating neuronal harm caused by ischemic stroke and various neurological disorders. N-acetylcysteine [45] and baicalin [46] have been reported to improve neurological function, reduce neuronal cell death, and alleviate brain injury by inhibiting ferroptosis. Based on this data, there have been limited studies that have documented ferroptosis and its associated molecular mechanisms in SAH.

Numerous studies have suggested that surgical trauma, concussion, stroke, and traumatic brain injury (TBI) have the potential to trigger neuroinflammation in the CNS [24, 47, 48]. Polarization of microglia plays an important role in the regulation of neuroinflammation, PPARγ activation can inhibit pro-inflammatory microglial responses, regulate microglial/macrophage M1/M2 polarization, and alleviate neuroinflammation [26, 49]. Excessive activation of microglia can result in neurotoxicity and damage to neurons, known as neuroinflammation [50]. According to Su [51], microglia were promptly triggered to transform into M1 and M2 microglia following disruptions in the microenvironment, contributing to both tissue harm and restoration. The current investigation revealed that OA significantly reduces the M1-like polarization of microglial/macrophage cells, while simultaneously enhancing M2-like polarization. As a result, it effectively mitigates brain damage following SAH. According to a recent investigation, OA can shield against MI/RI by diminishing the inflammatory reaction and impeding pyroptosis [34].

OA, baicalein-7-O-glucoside, was obtained from *Oroxylum indicum* (L.) Kurz, a traditional Chinese herbal medicine [52]. According to Sun [29], OA exhibits partial agonistic effects on PPARγ, leading to the activation of PPARγ transcriptional activation both in vitro and in vivo, and also possesses antioxidant properties. According to Huang [30], OA has the potential to relieve cell damage, possibly by inhibiting pyroptosis and exerting anti-inflammatory properties, then to hinder the growth of breast cancer cells through the stimulation of senescence mediated by ER stress. Our early animal experiments found that OA has the potential to enhance the neurological result in rat models following SAH. It is not clear what the role of the PPARγ agonist OA is in preventing ferroptosis in SAH, and it is also unclear if there are other mechanisms or unconventional pathways involved in ferroptosis. The current research demonstrated that OA mitigates neuronal harm by inhibiting ferroptosis following SAH in laboratory/animal models through the modulation of either the Nrf2/GPX4 or FSP1/CoQ10 signaling pathway.

Nrf2 is a crucial suppressor of ferroptosis as it controls the cellular antioxidant response, iron/heme metabolism, and lipid metabolism, and reduces electrophilicity [15, 38]. Moreover, when Nrf2 is activated, it can boost the quantities of cellular NADPH and glutathione, thereby enhancing iron storage. This is crucial in the prevention of ferroptosis, as stated in references [18, 38, 53]. Our prior investigation demonstrated that netrin-1 can safeguard hippocampal neurons and enhance neurological impairment through the regulation of the PPARγ/Nrf2-ARE pathway following SAH [17]. In the present investigation, we also found that the Nrf2 levels were decreased following SAH and restored with OA treatment, the possible molecular pathways through which Nrf2 controls the transcription of proteins, such as GPX4 and HO-1. Increasing evidence indicates the significance of the HO-1 and GPX4 pathways in ferroptosis [54–56]. The current investigation demonstrated that suppression of Nrf2 hindered the stimulation of GPX4, resulting in elevated lipid ROS levels, and GPX4-related ferroptosis depends on Nrf2 and that si-Nrf2 aggravates neuronal death after SAH. Furthermore, OA induced transcriptional activation of the FSP1 gene, which subsequently led to FSP1 activation in the neuronal nucleus. The FSP1 was then transferred to the plasma membrane, forming the antioxidant system that effectively prevents lipid peroxidation and ferroptosis. FSP1, a CoQ10 oxidoreductase, is directed to the plasma membrane through its N-terminal nutmeg acylation, the reduction of CoQ10 through NADH is facilitated at the cell’s outer surface, leading to the inhibition of CoQ10 activity and ultimately protecting ferroptosis [17, 57–59]. Interestingly, it was clarified that OA protects neuronal ferroptosis after SAH through the modulation of the Nrf2/GPX4 and FSP1/CoQ10 pathways. Nevertheless, the specific role regarding the control of FSP1 activation remains to be fully elucidated to ascertain its precise mechanism further.

## Conclusion

To summarize, the current study has offered empirical evidence that ferroptosis, triggered by GPX4 and FSP1, serves as a crucial cellular regulatory mechanism that could greatly impact EBI after SAH. For the initial occasion, we disclosed that OA can control ferroptosis through the Nrf2/GPX4 pathway and the CoQ10-FSP1 pathway. Additionally, we suggest a unique method for examining the biological effects of OA. Therefore, ferroptosis could potentially serve as a promising therapeutic approach for EBI following SAH, while OA holds significant clinical significance in the coming years and may emerge as a crucial prognostic biomarker for SAH individuals.

### Electronic supplementary material

Below is the link to the electronic supplementary material.

Supplementary Material 1

## Data Availability

All data used or evaluated during the present research are accessible following a valid request from the corresponding author.

## Abbreviations

CNS: Central nervous system
CVS: Cerebral vasospasm
EBI: Early brain injury
ECA: External carotid artery
ER: Endoplasmic reticulum
GSH: Glutathione
GPX4: Glutathione peroxidase 4
ICA: Internal carotid artery
ICH: Intracerebral hemorrhage
MDA: Malondialdehyde
Nrf-2: Nuclear factor erythroid 2-related factor 2
OA: Orexin A
ROS: Reactive oxygen species
RT-Qpcr: Real-time quantitative PCR
SAH: Subarachnoid hemorrhage
SD: Standard deviation
TNF-α: Tumor necrosis factor-alpha
