# Supplementary figures and images for: Oroxin A alleviates early brain injury after subarachnoid hemorrhage by regulating ferroptosis and neuroinflammation
Source: J Neuroinflammation. 2024 May 3;21:116. doi: 10.1186/s12974-024-03099-3 (PMC11069275; doi:10.1186/s12974-024-03099-3)

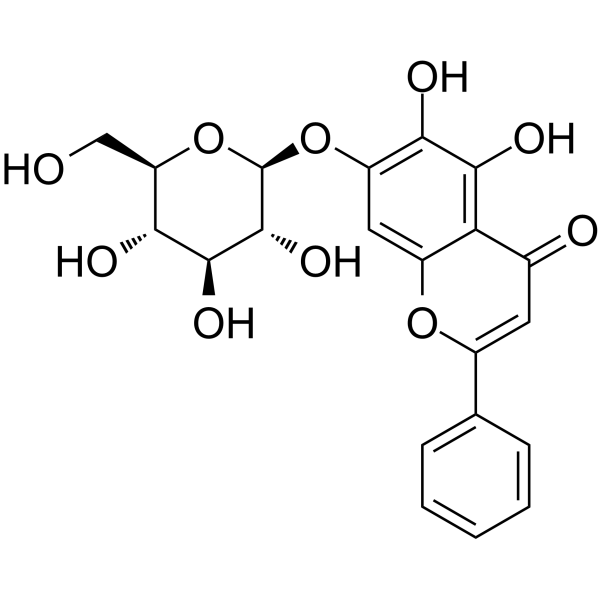

Supplement: Supplementary file 1 — Supplementary Material 1 [file 12974_2024_3099_MOESM1_ESM.gif]
